# Operational Challenges in Diagnosing Multi-Drug Resistant TB and Initiating Treatment in Andhra Pradesh, India

**DOI:** 10.1371/journal.pone.0026659

**Published:** 2011-11-02

**Authors:** Sarabjit S. Chadha, Sharath BN, Kishore Reddy, Jyothi Jaju, Vishnu PH, Sreenivas Rao, Malik Parmar, Srinath Satyanarayana, Kuldeep Singh Sachdeva, Nevin Wilson, Anthony D. Harries

**Affiliations:** 1 International Union against Tuberculosis and Lung Disease (The Union), South-East Asia Regional Office, New Delhi, India; 2 Office of the WHO-Representative in India, World Health Organization, New Delhi, India; 3 State TB Training and Demonstration Center, Irranuma, Hyderabad, India; 4 State TB Office, Directorate of Health, Government of Andhra Pradesh, Hyderabad, India; 5 International Union against Tuberculosis and Lung Diseases (The Union), Paris, France; 6 Central TB Division, Directorate General of Health Services, Ministry of Health and Family Welfare, Government of India, New Delhi, India; McGill University, Canada

## Abstract

**Background:**

Revised National TB Control Programme (RNTCP), Andhra Pradesh, India. There is limited information on whether MDR-TB suspects are identified, undergo diagnostic assessment and are initiated on treatment according to the programme guidelines.

**Objectives:**

To assess i) using the programme definition, the number and proportion of MDR-TB suspects in a large cohort of TB patients on first-line treatment under RNTCP ii) the proportion of these MDR-TB suspects who underwent diagnosis for MDR-TB and iii) the number and proportion of those diagnosed as MDR-TB who were successfully initiated on treatment.

**Methods:**

A retrospective cohort analysis, by reviewing RNTCP records and reports, was conducted in four districts of Andhra Pradesh, India, among patients registered for first line treatment during October 2008 to December 2009.

**Results:**

Among 23,999 TB patients registered for treatment there were 559 (2%) MDR-TB suspects (according to programme definition) of which 307 (55%) underwent diagnosis and amongst these 169 (55%) were found to be MDR-TB. Of the MDR-TB patients, 112 (66%) were successfully initiated on treatment. Amongst those eligible for MDR-TB services, significant proportions are lost during the diagnostic and treatment initiation pathway due to a variety of operational challenges. The programme needs to urgently address these challenges for effective delivery and utilisation of the MDR-TB services.

## Introduction

Globally, with an estimated annual incidence of more than half a million cases, Multi-Drug Resistant-Tuberculosis (MDR-TB) i.e. tuberculosis resistant to at least isoniazid and rifampicin, is a public health threat [1]. The Global Stop TB Strategy outlines and defines the programmatic management of drug resistant TB (PMDT) within National TB Programmes based on the principles of DOTS (Directly Observed Treatment Short Course). Timely identification of MDR-TB cases and prompt initiation of treatment is crucial to prevent the transmission of disease and reduce related high morbidity and mortality [2].

MDR-TB case-finding strategies vary, depending on the local epidemiological situation and capacity for quality assured diagnosis and treatment. In resource rich settings, all TB patients are tested with culture and Drug Susceptibility Testing (DST). However, in most resource limited settings, only patients considered to have an increased risk of MDR-TB are tested. Undiagnosed, untreated or improperly treated patients with MDR-TB are a source of ongoing transmission of resistant strains within the community, resulting in future added costs and mortality [3].

In India, with the highest burden of Tuberculosis globally, the prevalence of MDR-TB is estimated to be <3% amongst new cases and 14–17% amongst the re-treatment cases [4]; it is also estimated that ∼99,000 MDR-TB cases occur in the country annually [5]. To address the challenge of MDR-TB, the Revised National Tuberculosis Control Programme (RNTCP) of India has initiated MDR-TB services, at a sub-national level, in 2007 in a limited geographical area and is in the process of expanding these services, in a phased manner, to cover the entire country by 2012. Due to limited quality assured laboratory capacity the programme enrols only those patients identified to be at a high risk of MDR-TB (MDR suspects) for diagnostic assessment and subsequent treatment [6].

RNTCP has limited information on the proportion of MDR-TB suspects amongst TB patients on first line treatment within the programme, whether all these MDR-TB suspects are identified and undergo diagnostic assessment and whether all those diagnosed as MDR-TB are initiated on treatment according to the programme guidelines. We therefore decided to assess in a large cohort of TB patients registered for first-line anti-TB treatment i) the number and proportion who were MDR-TB suspects according to the programme definition ii) proportion of eligible MDR-TB suspects who underwent diagnosis (culture and DST) for MDR-TB and iii) the number and proportion of those diagnosed as MDR TB who were successfully initiated on MDR-TB treatment (within the programme).

## Methods

### Study Setting

The state of Andhra Pradesh (AP) has a population of ∼83 million with 23 administrative districts and 24 district TB control units. The State has an RNTCP accredited Culture and DST laboratory (Intermediate reference Laboratory), located in the State Training and Demonstration Center (STDC) at Hyderabad and has been performing culture and DST, using solid culture, for first line anti-TB drugs (SRHE) since June 2008. MDR-TB services were initiated in 4 districts of Andhra Pradesh (Hyderabad, Rangareddy, Nalgonda and Medak) in the Phase-1 in August, 2008. This study was conducted in these four districts.

### Treatment of Tuberculosis and definitions of MDR-Suspect and MDR-TB patient under RNTCP

TB patients diagnosed under the RNTCP were categorised as ‘new’ or ‘retreatment’ cases, initiated on first line treatment and registered in one of three categories of first line anti-TB regimens using standard case definitions recommended by WHO treatment guidelines [7], [8]. In brief, categories I and III are used for the treatment of ‘new’ TB cases and category II is used for the treatment of re-treatment cases (patients who have been previously treated with first line anti-TB drugs for at-least a month anytime in the past).

In all the three categories, treatment is administered to patients under the direct supervision of a DOT provider. All TB patients initiated on treatment are registered in a “Tuberculosis Register” maintained in the corresponding Tuberculosis Unit (1 Tuberculosis Unit ∼500,000 population). The Tuberculosis register is maintained and updated by supervisory staff called the Senior Treatment Supervisor (STS). The TB register contains the patients' basic demographic, clinical and treatment related information including details of the follow-up sputum examination.

**Figure 1:**
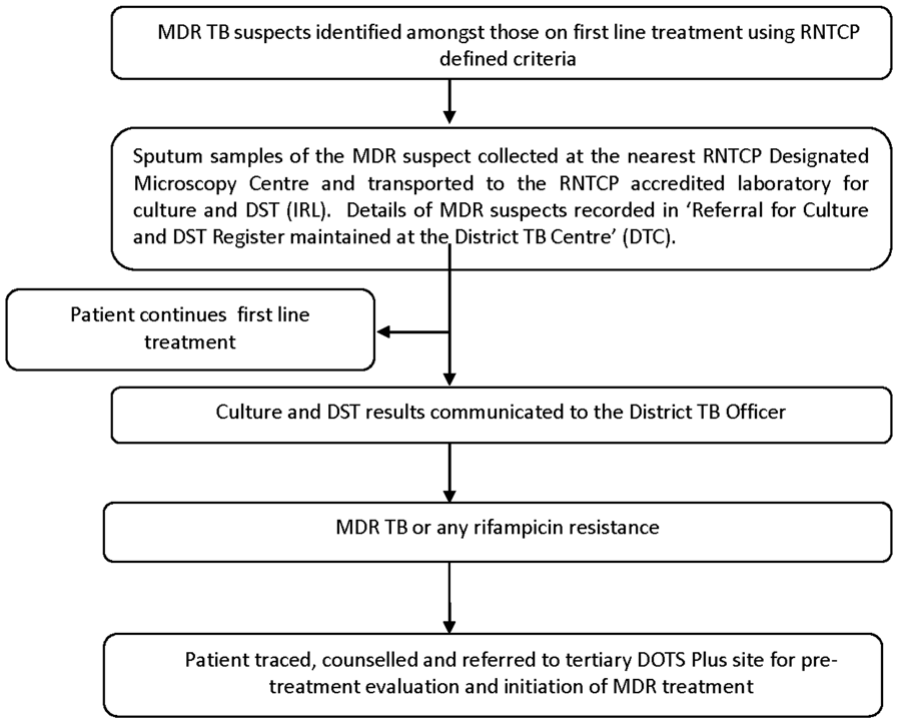
Diagnostic and treatment initiation pathway for Multi-drug resistant TB (MDR- TB) under RNTCP.

RNTCP DOTS Plus programme guidelines[6] defined an ‘MDR-TB suspect’ as: i) any TB patient who failed a category I or category III regimen ii) any category II patient who remained smear positive at the end of the fourth month of treatment or later, and iii) a smear positive contact of an MDR case regardless of prior anti-TB treatment. Patients who met this definition of MDR-TB suspect were to be identified and offered diagnostic services for MDR- TB as shown in **Figure 1**. The District TB Officer (DTO) is responsible for maintaining all the patient records and registers through RNTCP staff available at the DTC. The sputum is transported from the DMC to IRL under information to the DTO. The DTO also verifies from the previous treatment records whether the patient fulfils the criteria of MDR suspect.

### Study design, Study Population and sample size

The design was a retrospective record review study. All patients registered for treatment with first line anti-tuberculosis treatment during the period October 2008 to December 2009 within RNTCP in the four Phase-1 districts implementing DOTS-plus services in Andhra Pradesh (Hyderabad, Rangareddy, Nalgonda and Medak) were included in the study. This cohort included 23,999 patients with all forms of TB. The study was conducted in the month of February, 2011.

### Study variables and sources of data

Data variables and their source (within brackets) were: Number of TB patients registered (TB Register), their date of registration (TB Register), type of TB (TB Register), sputum smear status (TB Register), the results of follow-up sputum smear status (TB Register) and treatment outcomes (TB Register), number of MDR suspects (TB Register), number of MDR suspects referred for culture and DST (TB Register and Referral for culture and DST Register), number of MDR suspects diagnosed as MDR-TB (Culture and DST Register at the accredited laboratory), number of diagnosed MDR cases initiated on treatment (DOTS Plus TB Register maintained at the DOTS Plus site). .

### Data management and statistical analysis

Data from relevant records was extracted to a pre-structured questionnaire independently by two study investigators, cross checked for consistency, and all discrepancies were resolved by referring to the original records. Data was entered and analysed using Epidata statistical software package (version 3.1). Proportions have been used to summarise the various variables.

### Ethics

This study was a record review of routinely collected programme surveillance data for which approval from the Central TB Division, Ministry of Health and Family Welfare, Govt. of India and the State TB Cell, Directorate of Health Services, Government of Andhra Pradesh was obtained. The study protocol was also reviewed and approved by the Ethics Advisory Group of the International Union against Tuberculosis and Lung Disease.

## Results

There were 559 (2%) MDR-TB suspects (program defined) among 23,999 TB patients registered for treatment during the study period. For each of the five quarters under the study period (Oct 2008 to Dec 2009), the proportion of MDR-TB suspects amongst TB patients was almost similar varying from 2.0% to 3.0% (**Table 1**). The characteristics of these MDR-TB suspects is given in Table 2

**Table 1:** Multi-drug resistant TB (MDR-TB) suspects (as per RNTCP definition) in a cohort of patients in Andhra Pradesh.

Cohort	Number registered(n)	Number of MDR suspects(n) (%)	
Oct–Dec 2008	4472	98	2.2
Jan–Mar 2009	4824	143	3.0
Apr–Jun 2009	5332	108	2.0
Jul–Sep 2009	4709	116	2.5
Oct–Dec 2009	4662	94	2.0
**Total**	**23999**	**559**	**2.0**

**Table 2:** Characteristics of MDR-TB suspects in a cohort of patients in Andhra Pradesh (N = 559).

Characteristic	N	(%)
**Gender**		
Male	410	73
Female	149	27
**Age group**		
<15 Years	14	(3)
15–24 years	113	(20)
25–34 years	132	(24)
35–44 years	128	(23)
45–54 years	106	(19)
55–64 years	57	(10)
>64 years	7	(1)
**Treatment category**		
Cat-1	217	(39)
Cat-2	331	(59)
Cat-3	11	(2)
**Status of HIV**		
Negative	407	(73)
Positive	37	(7)
Unknown	115	(21)

**Figure 2:**
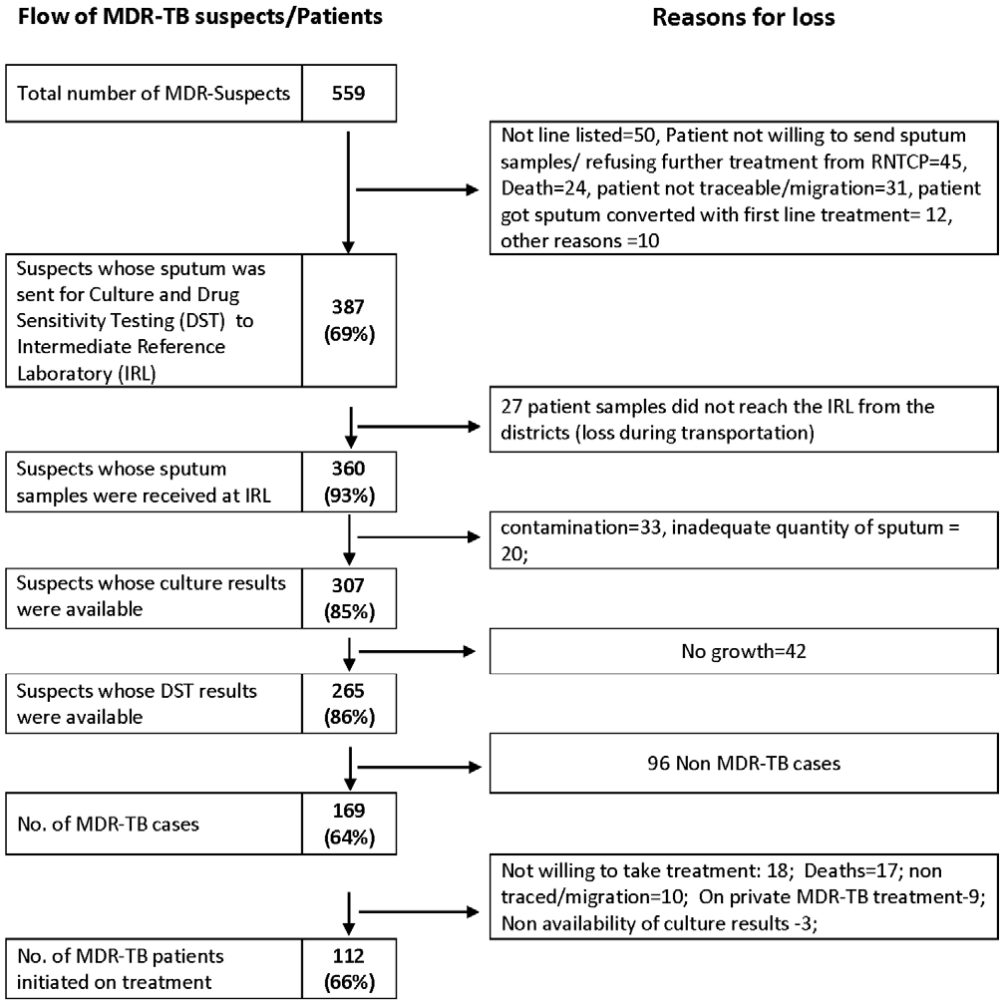
Loss of MDR-TB suspects along the diagnostic and treatment initiation pathway under RNTCP in Andhra Pradesh.

Out of 559 MDR-TB suspects, sputum was collected and transported from the periphery to the culture and DST laboratory in 387 (69%) cases of which 360 (93%) were received at the laboratory. The culture results were available for 307 (85%) of which DST results were available in 265 (86%). Among these 265 cases, 169 (64%) were diagnosed as MDR-TB. Of these 169 patients, 112 (66%) of the patients were initiated on MDR-TB treatment regimen under RNTCP. The flow of patients and the reasons for loss of patients at each step in the process of diagnosis and initiation of treatment is shown in **Figure 2**.

Overall, out of the 559 MDR-TB suspects, 307 (55%) underwent diagnosis for MDR-TB. 169 patients were diagnosed as MDR-TB of which 112 (66%) were initiated on MDR-TB treatment.

## Discussion

This is the first study which describes the flow of MDR-TB suspects along the diagnostic and treatment initiation pathway under RNTCP. About 2% of a large cohort of TB patients was classified as MDR-TB suspects, and this was similar in each of the five quarterly cohorts analysed. There was a fall off of patients at each stage of the referral and diagnostic process, resulting in only one third of MDR-TB suspects (programme defined) being diagnosed with MDR-TB and only 66% of diagnosed patients initiating treatment.

The study was operational in nature and relied on a review of registers in various locations. This study identifies three major operational challenges that are programmatically relevant and have had important programmatic implications.

The first is the significant patient loss during and after identification as MDR-TB suspects. It was observed that a significant number of MDR-TB suspects miss identification by the programme staff which calls for appropriate training and more intensive supervision. In some instances the programme staffs were line listing the suspects but this was not uniformly practiced. A fourth of the identified MDR-TB suspects refused to undergo diagnosis which highlights the need for expert counselling and motivation which is presently missing in the programme. Proper address verification and repeated retrieval efforts will help in reducing the number of patients who could not be traced. Deaths can be prevented in this group through steps taken to ensure early identification and diagnosis which may require a change in the existing criteria of failing a first line regimen to be eligible as a MDR-TB suspect to all TB patients who continue to be smear positive during follow up and finally to all TB patients on initiation of treatment. This will also require a rapid scale up of the laboratory capacity across the country.

The second is the loss in number of samples for which culture result should have been available. There is a need for an efficient and secure system to prevent loss during transportation. The high rates of contamination and culture negativity requires better collection of sputum samples and more robust laboratory procedures. Adoption of new molecular technologies like Line Probe Assay (LPA) and Nucleic Acid Amplification tests (NAAT) [9] which preclude the need for culture will also help in rapid diagnosis and avoid this loss.

The third is to ensure initiation of treatment for all diagnosed MDR-TB patients. In our present study, the extent of this loss was 34% attributable primarily to refusal for treatment (including those who had initiated treatment outside the programme). This could be due to the long delay in the availability of results, inability to travel to distantly located DOTS Plus sites for pre-treatment evaluation. This reiterates the need for deployment of rapid diagnostics and decentralisation of centres where treatment can be initiated. This will also help in early initiation of treatment which will prevent significant number of deaths in this group.

These three operational challenges are likely to be encountered by most TB control programmes in low and middle income countries that are initiating MDR-TB treatment services. Such challenges have also been noted in China [10] and these challenges are a result of not having decentralised rapid diagnostic and treatment initiation facilities. Care must be taken to ensure that the loss of patients is minimised by instituting adequate programmatic mechanisms.

In order to address these challenges RNTCP has instituted a series of corrective programmatic mechanisms. This includes formulating programme guideline that all sputum specimens should reach the designated culture and DST laboratory within 2 weeks of the patient being identified as an MDR-TB suspect and assigning the responsibility of monitoring this activity by a dedicated supervisor. This has resulted in instituting a mechanism for collection of sputum specimens as soon as the follow-up sputum smears are found positive at the DMC and are provided to the patient thus ensuring that they are transported within the stipulated time by a dedicated transportation mechanism. In order to reduce the time duration between receipt of specimens at the culture & DST laboratory and the declaration of results, the programme is introducing newer rapid diagnostic technology such as LPA in place of solid culture and DST. For patients who refuse MDR treatment primarily due to inability to travel to DOTS Plus sites, usually located at large distances, for pre-treatment evaluation and treatment initiation the programme provides incentives to cover the cost of the travel of the patient and one attendant. In addition, there is a provision for initiating treatment at the district level where facilities exist for pre-treatment evaluation. This study also provides baseline information that can be compared with results from subsequent changes in programme design or definitions. In addition, the RNTCP is also undertaking intensive and follow up trainings of the staff on identification of MDR-TB suspects and counselling of patients to prevent drop out at various stages.

### Limitations of the study

The study being retrospective in nature and has its inherent limitations of record review studies. If the records maintained at various levels were not updated periodically or were incorrect for any reason, then this has the potential to affect the study results. The programme is however, supervised and monitored regularly from various staff and includes periodic data validation, hence the likelihood of any recording errors are minimal [11]. Secondly, in order to fully understand the operational challenges, both provider and patient perspectives are needed. This study however provides information on the operational aspects from the programme perspective and not from the patient perspective.

### Conclusion

In a cohort of TB patients in one of the sites in India, there were 2% MDR-TB suspects (programme defined), about 70% of these underwent diagnosis, 30% were found to be MDR-TB of which 66% were initiated on MDR-TB treatment. There is an urgent need to ensure that operational challenges that are resulting in the loss of patients during the MDR-TB diagnostic and treatment pathway are adequately addressed by corrective mechanisms initiated by national programmes.
